# Characterization of Neurogenic Potential of Dental Pulp Stem Cells Cultured in Xeno/Serum-Free Condition:* In Vitro* and* In Vivo* Assessment

**DOI:** 10.1155/2016/6921097

**Published:** 2016-09-05

**Authors:** Jieun Jung, Jong-Wan Kim, Ho-Jin Moon, Jin Young Hong, Jung Keun Hyun

**Affiliations:** ^1^Institute of Tissue Regeneration Engineering, Dankook University, Cheonan, Republic of Korea; ^2^Department of Nanobiomedical Science and BK21 PLUS NBM Global Research Center for Regenerative Medicine, Dankook University, Cheonan, Republic of Korea; ^3^Department of Restorative Dentistry & Endodontics, College of Dentistry, Dankook University, Cheonan, Republic of Korea; ^4^Department of Rehabilitation Medicine, College of Medicine, Dankook University, Cheonan, Republic of Korea

## Abstract

Neural stem cells (NSCs) have a high potency for differentiation to neurons and glial cells for replacement of damaged cells and paracrine effects for the regeneration and remyelination of host axons. Dental pulp is known to have a potential to differentiate into neural-like cells; therefore, dental pulp may be used as an autologous cell source for neural repair. In this study, we selectively expanded stem cells from human dental pulp in an initial culture using NSC media under xeno- and serum-free conditions. At the initial step of primary culture, human dental pulp was divided into two groups according to the culture media: 10% fetal bovine serum medium group (FBS group) and NSC culture medium group (NSC group). In the NSC group relative to the FBS group, the expression of NSC markers and the concentrations of leukemia inhibitory factor, nerve growth factor, and stem cell factor were higher, although their expression levels were lower than those of human fetal NSCs. The transplanted cells of the NSC group survived well within the normal brain and injured spinal cord of rats and expressed nestin and Sox2. Under the xeno- and serum-free conditions, autologous human dental pulp-derived stem cells might prove useful for clinical cell-based therapies to repair damaged neural tissues.

## 1. Introduction

Stem cell-based therapies using neural stem cells (NSCs) are considered to be one of the most promising strategies for treatment of the lesions of the central and peripheral nervous systems [1, 2]. Endogenous NSCs are known to exist within the adult brain and even in the spinal cord; unfortunately, their capacity for neural regeneration following stroke or spinal cord injury in adults is very limited, as they are difficult to stimulate within* in vivo* microenvironments [2, 3]. Exogenous NSCs from embryos or fetus which have been shown to be effective for neural regeneration [4, 5] still have immunological, ethical, and political problems [6]. Recently developed induced pluripotent stem cells (iPSCs) and direct reprogrammed NSCs [7–9] also have potential risks of viral integration, tumor formation, and genomic instability which remain hurdles to clinical translation [10]. Among other concerns about the clinical application of stem cells, there are animal components such as fetal bovine serum (FBS) which can incur the risk of transmitting pathogens and immune responses to recipients [11, 12].

Various cell types including endothelial cells, fibroblasts, odontoblasts, mesenchymal stem cells (MSCs), and neural cells are contained in dental pulp easily obtainable from human adults and infants. Stem cells within dental pulp (i.e., dental pulp stem cells: DPSCs) have a high potential for proliferation and differentiation into neural-like cells and as such might be a good source for neural regeneration [13]. Previous studies in fact have demonstrated successful differentiation of human dental pulp-derived stem cells into neural-like cells in both* in vitro* and* in vivo* conditions [14–17], and other studies, moreover, have found that neural-like cells can effectively promote functional improvements in rodent nerve injury models [18, 19].

The aim of this study was to establish a method for isolation and expansion of stem cells from human dental pulp under xeno- and serum-free conditions as well as investigate whether these cells express key neural genes after transplantation into neural tissues of rats.

## 2. Materials and Methods 

### 2.1. DPSC Isolation and Culture

This study was approved by the Institutional Review Board of Dankook University Dental Hospital in Korea (approval number H-1304/005/003). Normal third molars were extracted and collected from three healthy patients (aged 22-23 years). One oral surgeon gently separated the dental pulp from the periodontal ligament and gingival tissue without contamination, subsequently cutting it into small pieces for incubation in mixed collagenase type I/dispase solution for 1 hour. Large aggregates and debris were removed by passing the cells through a 70-*μ*M strainer. The obtained single cells were divided equally into two groups: a control group and an experimental group. In the control group (FBS group), cells were suspended and cultured with FBS media: Dulbecco's modified Eagle's medium/Ham's F12 medium (DMEM/F12) supplemented with 10% FBS and 1% penicillin/streptomycin (P/S, all from Invitrogen). In the experimental group (NSC group), cells were cultured in NSC media: DMEM/F12 supplemented with 1% P/S, 20 ng/mL basic fibroblast growth factor (bFGF, R&D Systems), 20 ng/mL epidermal growth factor (EGF, R&D Systems), N2 (Invitrogen), and B27 (Invitrogen), under the xeno- and serum-free conditions. All of the cells of both groups, FBS and NSC, were used at passage 5 for later analysis. Two control cell lines were used to compare the cell metabolic activity and quantitative real-time polymerase chain reaction (qRT-PCR) analysis: bone-marrow-derived MSCs (BM-MSCs, from ATCC) which were cultured in DMEM/F12 medium supplemented with 10% FBS, 1% P/S, and human fetal brain-derived NSCs (hNSCs) (from Professor Cho) which were cultured in DMEM/F12 medium supplemented with 1% P/S, bFGF (20 ng/mL), leukemia inhibitory factor (10 ng/mL, Sigma), and N2.

### 2.2. Neural Differentiation of DPSCs

For neural differentiation, plastic film-surfaced coverslips were placed on a 24-well plate and coated with laminin and fibronectin at 4°C overnight after coating with type I and IV collagen (all 10 *μ*g/mL) for 1 h at room temperature (RT). The cells at passage 5 in FBS and NSC group were seeded at 2 × 10^4^ cells/well and cultured in DMEM/F12 medium supplemented with 1% P/S, N2, B27, retinoic acid (100 nM), sonic hedgehog (100 ng/mL), brain-derived neurotrophic factor (10 ng/mL), glial-cell-derived neurotrophic factor (10 ng/mL), and insulin-like growth factor-1 (10 ng/mL) for 2 weeks and subsequently supplemented with N^6^,2′-O-dibutyryladenosine 3′,5′-cyclic monophosphate (dbcAMP, 1 mM) and forskolin (10 *μ*M) for 1 week.

### 2.3. MTT Assay

To determine the cellular metabolic activity, 3(-4,5-dimethylthiazol-2-yl)2,5-diphenyltetrazolium bromide (MTT) assay was performed. Cells in FBS or NSC groups or BM-MSCs were seeded at 2 × 10^3^ concentrations in a 96-well plate and cultured for 1 to 5 days in appropriate media. Then, the cells were dissolved with 100 *μ*L DMSO after 2-hour incubation with MTT (0.5 mg/mL) and measured by spectrophotometry (OD 570 nm, Bio-Rad Laboratories). The values were expressed as fold changes. The analysis was performed in three independent experiments using three separate wells at each time point.

### 2.4. Total RNA Isolation

The cells were seeded at 2 × 10^6^ concentrations on a 100 mm culture plate and then lysed in TRIzol (Invitrogen) three days later. Total RNA was extracted with chloroform, precipitated with isopropanol, washed in 75% ethanol, and dissolved in RNase- and DNase-free distilled water (Invitrogen). The RNA concentration was quantified by UV spectrophotometry (NanoDrop Technologies) at 260 nm. The total RNA was stored at −80°C after extraction.

### 2.5. qRT-PCR Analysis

Complementary DNA (cDNA) was synthesized with 80 ng of RNA by 20 *μ*L reverse transcription reaction using the High-Capacity RNA-to-cDNA Kit (Applied Biosystems). The qRT-PCR of FBS and NSC groups (*n* = 3 each) was performed in StepOnePlus (Applied Biosystems) using the SYBR® Green PCR master mix (Applied Biosystems) containing 0.5 mM of each primer and 2 *μ*L of template in 20 *μ*L of final volume. The sequences of the forward and reverse primers are listed in Table 1. The triplicate reaction for the amplification conditions proceeded as follows: 5 minutes at 95°C, with 25–32 cycles at 94°C for 30 seconds, at 55–60°C for 1 minute, and at 72°C for 1 minute. The expression level of each gene was normalized to 18s ribosomal RNA.

### 2.6. Fluorescence-Activated Cell Sorting (FACS) Analyses

The FACS analysis was performed on cells seeded at 2 × 10^6^ concentrations in a 100 mm dish after 3 days of culture. FBS and NSC medium cells were stained for 1 hour with the prescribed antibodies (listed in Table 2) and appropriate isotype controls as per the manufacturer's instructions. After being washed twice in phosphate-buffered saline (PBS), the cells were analyzed using FACSCalibur and CellQuest software (BD Biosciences).

### 2.7. Multiplex Supernatant Cytokine Assay

For the purposes of a cytokine assay, 2 × 10^4^ cells in FBS or NSC groups (*n* = 3) were seeded in a 24-well plate with 0.2 mL of DMEM-F12 supplemented with 1% penicillin/streptomycin (P/S) media, and the individual supernatants were harvested at 24 hours of culture. Cell-supernatant samples of 50 *μ*L were combined with coated beads using the MILLIPLEX™ MAP kit (Millipore) and analyzed. DMEM-F12 supplemented with 1% P/S was used both as a control and as a diluent for the standard samples. After incubation and washing instances, beads from the wells were resuspended in a 125 *μ*L cuvette of the Luminex apparatus. An acquisition gate was set between 7500 and 13,500 for doublet discrimination; the sample volume was 75 *μ*L, and 100 events/region were acquired. To obtain the concentration values, raw data (mean fluorescence intensity) from all of the bead combinations tested were analyzed using Master Plex QT3.0 quantification software (MiraiBio Inc.).

### 2.8. *In Vivo* Transplantation

To investigate the cell survivability under the* in vivo* condition, cells were transplanted into the neonatal brain (2 days, *n* = 3) and injured spinal cord (12 weeks, *n* = 3) of Sprague-Dawley (SD) rats. All of the animal care and surgical procedures were approved by the Institutional Animal Care and Use Committee of Dankook University in Korea (approval number DKU-12-019) and conformed to the ARRIVE guidelines. The contusion injury was applied to the T9 spinal cord level using the Infinite Horizon impactor (IH-400, Precision Systems and Instrumentation) as previously described [20]. Cells of both groups, NSC or FBS medium cells (5 × 10^5^ cells in 5 *μ*L of PBS), were transplanted into the cortex region at first, and then NSC medium cells were transplanted into the epicenter of the injured spinal cord at 9 days after contusion injury at a rate of 1 *μ*L/min using a Hamilton syringe (Hamilton Company). Cyclosporine A (Cipol Inj™, Chong Kun Dang Pharmaceutical Corp.) was administered subcutaneously at 10 mg kg^−1^ day^−1^ until the animals were sacrificed.

### 2.9. Immunofluorescence Staining

Cells were fixed in 4% paraformaldehyde (PFA) for 30 minutes at RT and washed with PBS 3 times. The rats were perfused and the brains removed 1 week after cell transplantation. The removed brain and spinal cord tissues were postfixed, immersed, and then embedded, whereupon coronal sectioning of the brain tissue and sagittal sectioning of the spinal cord tissue were performed serially. The prepared cell and tissue samples were incubated with primary antibody as listed in Table 2. Following incubation, the slides were washed with PBS and incubated with fluorescent-dye-conjugated secondary antibodies and stained with 4′,6-diamidino-2′-phenylindole dihydrochloride (DAPI) staining. Images were taken using a confocal microscope (Carl Zeiss Inc.).

### 2.10. Statistical Analysis

Statistical analyses were performed using PASW Statistics 18 (SPSS Inc., Chicago, IL, USA). The Shapiro-Wilk test was conducted to reveal the normal distribution of all quantitative data from MTT assay, qRT-PCR, and cytokine assays from* in vitro* studies, and Levene's test was conducted for homogeneity of variance of fold changes from MTT assay. According to the result, two-way repeated-measures ANOVA (cell types and time point) was performed to compare the fold changes from MTT assay among FBS and NSC groups and BM-MSC control, and individual comparisons were made at each time point by one-way ANOVA with Welch statistic and Games-Howell post hoc test. An independent *t*-test was performed to compare data from qRT-PCR and cytokine assays between FBS and NSC groups. *P* value less than 0.05 was considered significant.

## 3. Results

### 3.1. Morphology and Cellular Metabolic Activity of DPSCs Cultured under Different Media Conditions

**Figure 1:**
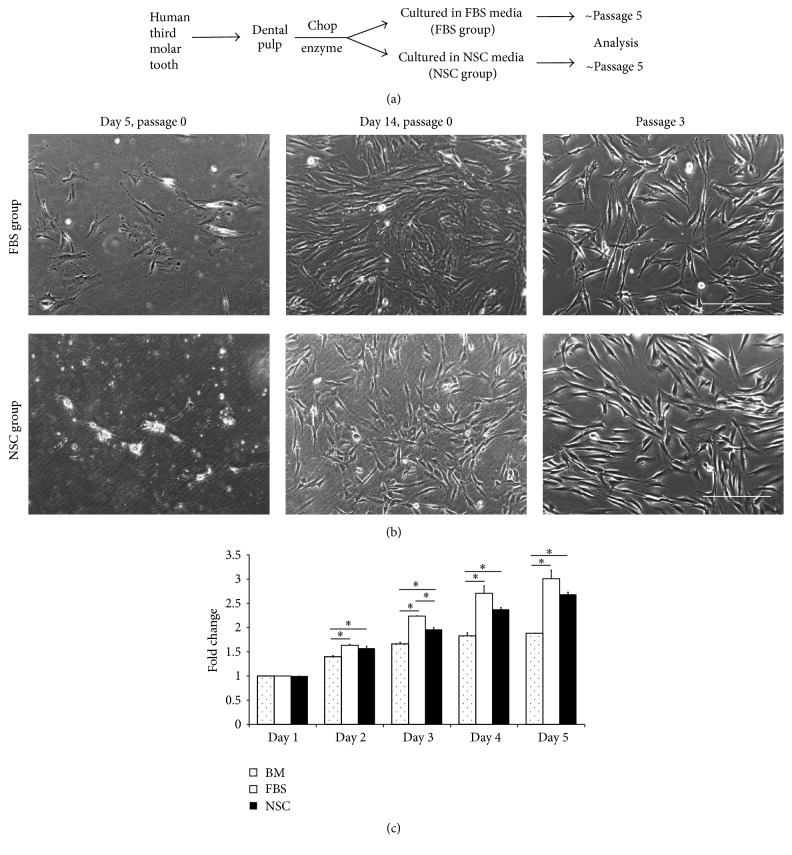
Strategy for derivation of neural stem-like cells from dental pulp of extracted human third molar. (a) Experimental scheme for classification of fetal bovine serum (FBS) and neural stem cell (NSC) groups. (b) Comparison of primary cell expansion and morphology between FBS and NSC medium cells. Scale bar = 100 *μ*m. (c) MTT assay to measure cellular metabolic activities of FBS medium cells, NSC medium cells at passage 5, and human bone-marrow-derived mesenchymal stem cells (BM-MSCs). The error bars represent the standard deviation of mean from three independent experiments from one donor (FBS and NSC groups) or one cell line (BM). Two-way repeated-measures ANOVA (Greenhouse-Geisser) revealed significant effects of day, cell group, and day × cell group interaction (*P* < 0.05). The asterisk (*∗*) indicates significant difference in comparison with BM-MSC at each time point according to one-way ANOVA and Games-Howell post hoc test (*P* <  0.05).

**Figure 2:**
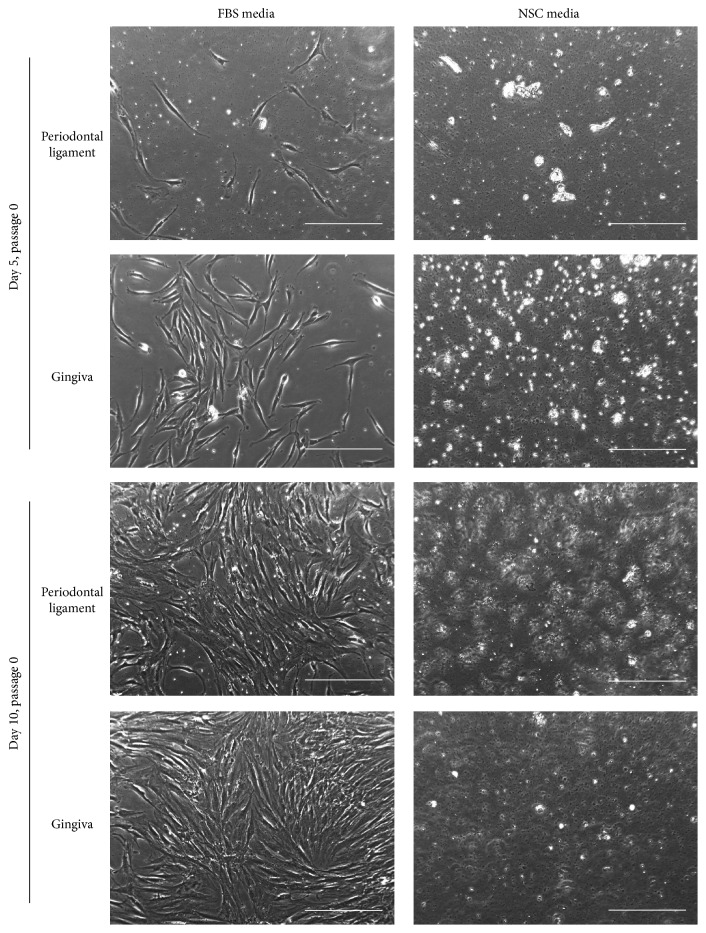
Primary cell adherent culture and morphology of periodontal ligament and gingival cells in FBS media or NSC media from day 5 to day 10. Scale bar = 100 *μ*m.

The cells from human dental pulp were firstly passaged at 1 week for FBS group and 2 weeks for NSC group after isolation (Figure 1(a)). Although the first passaging of the cells of NSC group required more time than for FBS medium cells (Figure 1(b)), we could obtain many strongly proliferative cells under NSC media condition (Figures 1(b) and 1(c)). At passage 3, FBS medium cells exhibited a rough surface and flattened fibroblast-like morphology, whereas NSC medium cells manifested a smooth surface and more convex shape (Figure 1(b)); non-pulp-tissue-derived cells, for example, from the periodontal ligament and gingiva, however, failed to grow in the NSC media (Figure 2). The MTT assay revealed that NSC medium cells' metabolic activity was similar to that of FBS medium cells during the initial 5-day culture period except for day 3 and was significantly higher than that of the human BM-MSCs (Figure 1(c)). These data indicate, significantly, that highly proliferative cells can be obtained from human dental pulp in a serum-free NSC medium from the initial culture stage.

### 3.2. Comparison of Marker Expressions between FBS and NSC Medium Cells

**Figure 3:**
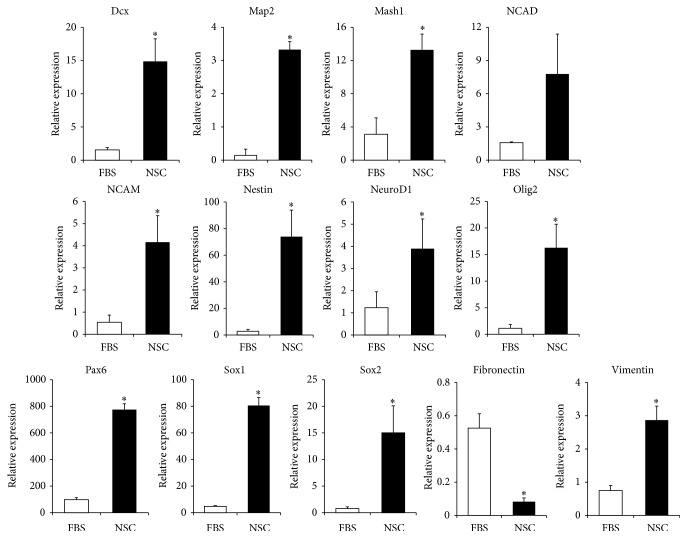
Comparison of marker expressions between FBS group (FBS, white bar) and NSC group (NSC, black bar) at passage 5. qRT-PCR was applied for measurement of NSC or neuronal markers (Dcx, Map2, Mash1, NCAD, NCAM, nestin, NeuroD1, Olig2, Pax6, Sox1, and Sox2), MSC marker (fibronectin), and both NSC and MSC markers (vimentin) in the FBS and NSC groups. The results are displayed relative to the expressions in FBS medium cells via calculation using the ΔΔCT and 2-(ΔΔCT) methods and normalized to 18s ribosomal RNA expression, and the error bars represent the standard deviation of mean from three donors in each group (*n* = 3). The asterisk (*∗*) indicates significant difference in comparison with the FBS group according to the independent *t*-test (*P* < 0.05).

The qRT-PCR revealed that the NSC markers, including doublecortin (Dcx), microtubule associated protein 2 (Map2), Mash1, neural cell adhesion molecule (NCAM, also known as CD56), nestin, neuronal differentiation 1 (NeuroD1), paired box protein 6 (Pax6), sex-determining region Y-box 1 (Sox1), Sox2, NSC and glial-cell marker including oligodendrocyte transcription factor-2 (Olig2), and vimentin, which is a marker of both NSC and MSC, were all expressed significantly higher in NSC group than in FBS group, whereas fibronectin, which is known as an MSC marker, was expressed significantly lower in NSC group than in FBS group (Figure 3). And individual expression patterns of FBS and NSC groups were shown in Supplemental Figure 1, in Supplementary Material available online at http://dx.doi.org/10.1155/2016/6921097, with the expression patterns of BM-MSC and fetal brain-derived NSCs (Supplemental Figure 1), and the expression level of most of the NSC markers in NSC group seemed to be lower than in human fetal brain-derived NSCs.

**Figure 4:**
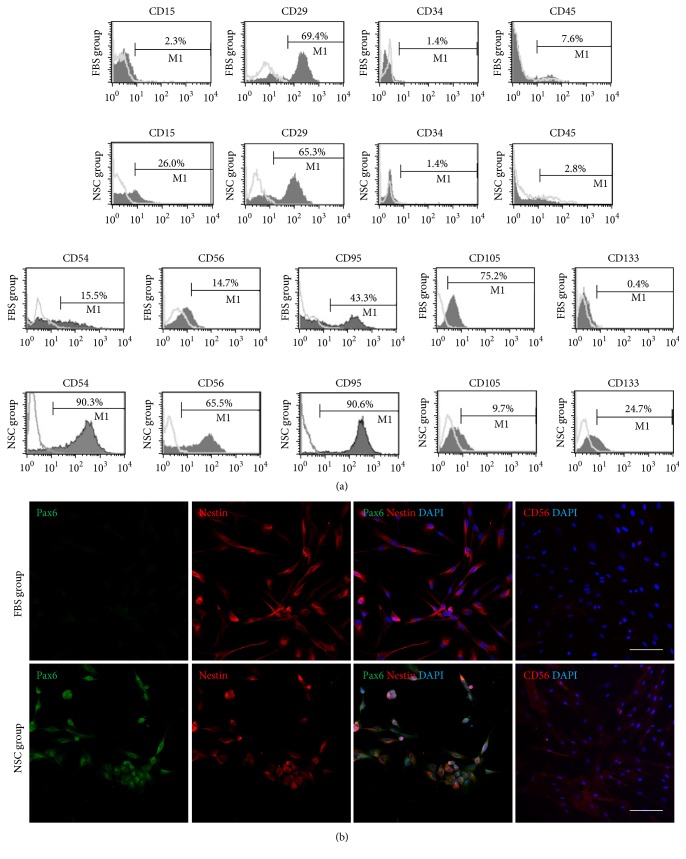
FACS analysis and immunocytofluorescence of FBS and NSC groups. (a) Expression of surface markers in FBS and NSC groups at passage 5 as analyzed by FACS. The NSC markers (CD15, CD29, CD54, CD56, and CD95), MSC marker (CD105), and hematopoietic markers (CD34, CD45, and CD133) in the FBS and NSC medium cells. (b) Immunocytofluorescence findings of FBS and NSC medium cells at passage 5. The cells were stained with Pax6, nestin, and CD56. Scale bar = 100 *μ*m.

Next, NSC- and MSC-specific surface markers were analyzed by FACS. In NSC group, the CD15-, CD54-, CD56-, CD95-, and CD133-positive cell proportions were much higher than those in FBS group. By contrast, the expression level of the MSC marker CD105 was superior in FBS group in comparison to the NSC group. The hematopoietic stem cell markers CD34 and CD45, meanwhile, were expressed negatively in both FBS and NSC medium cells (Figure 4(a)). As demonstrated in Figure 4(b), the immunocytochemical results for Pax6 were consistent with the qRT-PCR data for FBS and NSC groups, though nestin was similarly stained in both groups. These data indicate that the cells cultured in xeno- and serum-free NSC media from the initial step of primary culture showed higher NSC features compared with the cells cultured in the FBS-containing media.

### 3.3. Factors Secreted from FBS and NSC Medium Cells and Neural Differentiation

**Figure 5:**
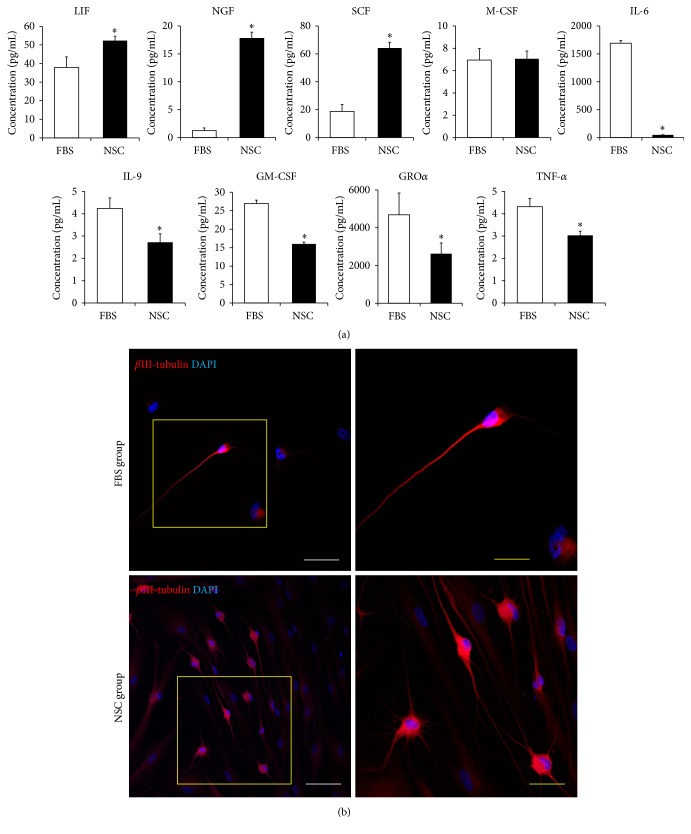
Growth factors, cytokine-secretion capacity, and neural differentiation potency. (a) Multiplex supernatant cytokine assay in FBS group (FBS, white bar) and NSC group (NSC, black bar) at passage 5. The error bars represent the standard deviation of mean from three donors in each group (*n* = 3). The asterisk (*∗*) indicates significant difference in comparison with the FBS group according to the independent *t*-test (*P* < 0.05). (b) Immunocytofluorescence findings of FBS and NSC medium cells after 3-week procedure for differentiation to neural-like cells. *β*III-Tubulin positive cells were stained in red, and nuclear counterstaining was performed with DAPI (blue). White scale bars = 100 *μ*m; yellow scale bar = 50 *μ*m.

The present multiplex supernatant human cytokine assay was performed to identify the factors secreted from FBS and NSC medium cells. In the cell supernatant of NSC group, the cytokines involved in self-renewal and differentiation of NSC, such as leukemia inhibitory factor (LIF), nerve growth factor (NGF), and stem cell factor (SCF) [21], were significantly more evident (*P* < 0.05) than in FBS group; meanwhile, the cytokines mainly involved in inflammation, such as interleukin-6 (IL-6), IL-9, granulocyte-macrophage colony-stimulating factor (GM-CSF), growth-regulated oncogene *α* (GRO*α*), and tumor necrosis factor *α* (TNF-*α*) [22], were significantly less abundant (*P* < 0.05) (Figure 5(a)).

Further, the neural differentiation potential of FBS and NSC medium cells was investigated, to which end *β*III-tubulin-positive cells were examined 3 weeks after neural differentiation (Figure 5(b)). Before neural differentiation, no clear *β*III-tubulin-positive cells were found in either FBS or NSC groups (Supplemental Figure 2). Three weeks after the differentiation process, we found that *β*III-tubulin-positive cells were visible in both FBS and NSC groups; however, the number of positive cells was higher in NSC group than in FBS group (Figure 5(b)). The data obtained indicated that NSC medium cells secreted multiple growth factors and stem cell-niche-related cytokines at higher levels than did FBS medium cells, thereby demonstrating, via manifested bipolar and multipolar morphologies, their neural differentiation potential.

### 3.4. *In Vivo* Transplantation of FBS and NSC Medium Cells into Rat CNS

**Figure 6:**
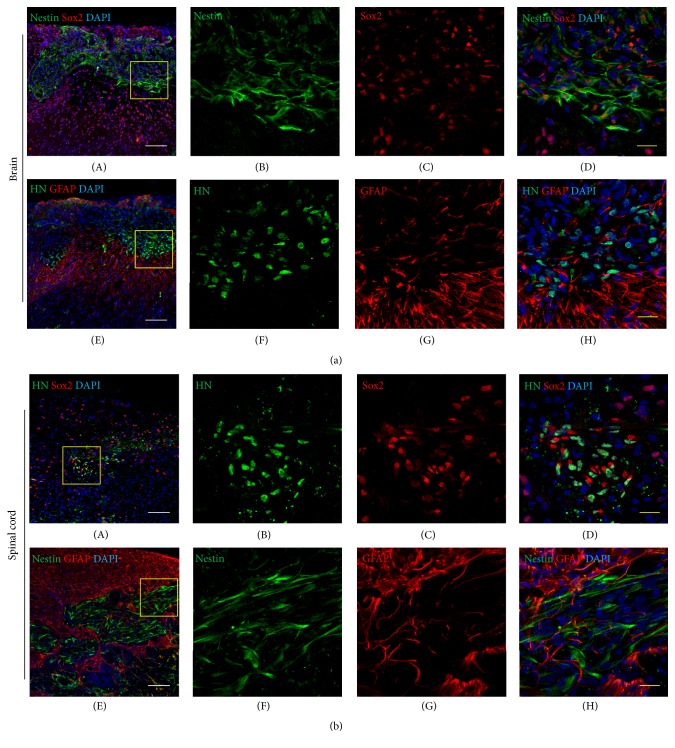
Immunohistofluorescence findings of the neonatal brain (a) and injured spinal cord tissues (b) of rats at 1 week after transplantation of NSC medium cells. The tissues were stained with human nuclei (HN, green, (a)(F) and (b)(B); GFAP, red, (a)(G) and (b)(G); nestin, green, (a)(B) and (b)(F); and Sox2, red, (a)(C) and (b)(C)), and nuclear counterstaining was performed with DAPI (blue). (a)(B–D), (a)(F–H), (b)(B–D), and (b)(F–H) show magnified images of yellow-line boxes in (a)(A), (a)(E), (b)(A), and (b)(E) images, respectively. White scale bars = 100 *μ*m; yellow scale bar = 20 *μ*m.

One week after transplantation of NSC medium cells, the transplanted cells had survived well within the brain and injured spinal cord and also had successfully expressed NSC markers (Figure 6). The engrafted NSC medium cells expressed Sox2, which were costained with nestin (Figure 6(a)) or human nucleus (Figure 6(b)); however, their staining intensity was lower than Sox2-positive host cells (Figure 6). We also transplanted FBS medium cells within the normal neonatal brain of rats, and transplanted cells survived with a small amount within the transplanted site 1 week after transplantation (Supplemental Figure 3). These data reflect NSC medium cells' favorable indications for survival and integration within the rat CNS.

## 4. Discussion 

Dental pulp, which is cell-rich soft tissue generated in the cranial neural crest during tooth development, contains heterogeneous populations such as odontoblast, fibroblast, pericyte, and neural cells as well as stem cells persisting in the adult dental pulp niche [23]. For culturing of DPSCs, most studies have utilized animal-serum-containing media to obtain sufficient cells during primary culturing, even for research on neural regeneration [14, 17]. However, serum is more suitable for MSC than for NSC growth; furthermore, animal serum incurs safety risks due to the possibility of the transmission of pathogens such as prions, viruses, and zoonoses. Also, animal-serum proteins can induce immunogenic reactions, which might reject transplanted cells even if autologous [12]. In light of these factors, establishment of a xeno-free culture system is required for any cell therapeutic strategies. In the present study then, in order to exclude animal components and to inhibit the expansion of MSC or fibroblasts within dental pulp, we strictly avoided the use of synthetic-, human-, or other animal-origin serums for the expansion of stem cells. Notwithstanding the many successful studies that have already investigated DPSC properties, the present investigation, to our knowledge, is the first to undertake the selective expansion of neural stem-like cells from human dental pulp without any exposure of animal serum from the initial step of primary culture, an approach that is highly advantageous from the clinical application safety standpoint. Although a previous study revealed the expansion abilities of DPSCs in a serum-free medium, the cells were cultured with 10%-FBS-supplemented medium on the first day before culturing them in the serum-free medium [23].

Herein, we hypothesize that stem cells can be expanded among the heterogeneous cells of dental pulp in serum-free NSC media from the initial step of primary culture. In the present study accordingly, two different culture conditions, FBS media and NSC media, were applied to dental pulp that had been divided from three independent donors. The results showed that the cells were greatly expanded in the NSC media until at least the 10th passage, though further study will be needed to determine the exact role in neural repair of NSC media. Significantly nonetheless, we found that no cells had grown from other dental tissues such as periodontal ligament- or gingival-derived tissues in the serum-free NSC media, whereas, in the animal-serum-containing media, the cells, as already demonstrated in relevant earlier studies, were well expanded (Figure 2) [24, 25]. These differences between dental pulp and other dental tissues might be caused by the diversity of cell types; certainly, the results tell us that dental pulp, as opposed to other oral-origin tissues, is favorable for obtainment of responsive cells under the neural basal condition. In further results, whereas the NSC medium cells only slowly expanded in the initial step of primary culture, they acquired a high growth capacity after adaptation to* in vitro* culture system. At the 5th passage, we investigated, by FACS analysis, whether cell surface markers were expressed differently in FBS and NSC medium cells. Surface markers act as essential compounds and receptors for various cellular metabolic processes such as proliferation and differentiation. Our data revealed, in fact, that a large number of DPSCs expressed the MSC marker when they were cultured in 10%-FBS-supplemented medium; however, the NSC markers were dominantly expressed when they were expanded in the NSC medium, even though the CD29-positive populations in the respective groups were similar. These marker patterns were shown in the qRT-PCR and immunofluorescence results concurrently with the FACS data. Overall, these data support the hypothesis that neuroectodermal origin human DPSCs presenting the typical profiles of NSC markers for gene and protein expression can be selectively expanded in xeno- and serum-free NSC media.

For regulation of the environmental niche for stem cells' survival and differentiation, paracrine effects are important. Previous studies analyzed the cytokine release profiles of DPSCs during osteo/odontogenic differentiation [26] or secretomes in the conditioned medium from stem cells from human exfoliated deciduous teeth [27]; however, there has been no study on NSC-related secretomes on DPSCs. In the current study, because secretion of cytokines related to self-renewal and tissue-repair and immunomodulatory factors are important for stem cell transplantation, we evaluated cytokines known to be secreted by NSCs and to affect neural activity [28–30]. In the results, cytokines known to be secreted by NSCs were highly contained in the supernatant of NSC medium cells, suggesting the possibility of neuroregenerative activities with paracrine effects. We additionally confirmed the neural differentiation potential (Figure 5(b)) and* in vivo* experiments, as an a priori study, revealed the survival ability of dental pulp-derived NSC medium cells within the rat CNS, specifically the normal brain and injured spinal cord (Figure 6). However, the expression level of NSC markers in NSC medium cells, as shown in qRT-PCR and immunohistochemistry, seemed to be lower than endogenous neural stem cells, and further* in vivo* researches will be needed to clarify the potential usefulness of human DPSC-derived NSC medium cells to promote functional restoration of CNS lesions.

Taken together, our investigations and results would seem to confirm the hypothesis that human dental pulp tissue contains stem cell populations applicable to neuroregenerative medicine. Optimization of cell-isolation and culture methods for such application, therefore, is called for. Nonetheless, small number of samples, which were obtained from only three subjects, might raise power issues, and future studies are required for elucidation of the potencies for differentiation to specific types of neural cells, for the therapeutic function on neural injury over the long term, and also for derivation of a more time-efficient method of initial expansion for safer application of NSC medium cells.

## 5. Conclusion

Here, we identified a novel method for safe and reliable cell expansion of stem cells from dental pulp in an initial culture using NSC media using, for the first time, xeno- and serum-free culture systems. This study may help pave the way for the clinical translation using cell-based therapies to repair and regenerate damaged neural tissues.

## Supplementary Material

Supplemental Figure 1. qRT-PCR measurements of NSC and MSC markers in the individual FBS (F1, F2, and F3, white bars) and NSC (N1, N2, and N3, black bars) groups at passage 5, and human BM-MSC (BM, dotted bar) and human fetal neural stem cells (FN, dashed bar) as controls. Supplemental Figure 2. Immunocytofluorescence findings of FBS and NSC medium cells at passage 5 just before the procedure for differentiation to neurons. Supplemental Figure 3. Immunohistofluorescence findings of rat brain tissues at 1-week post-transplantation of FBS medium cells.

## Figures and Tables

**Table 1:** Sequence of primers used for quantitative real-time PCR in this study.

Gene	Sequence
Dcx	F: 5′-AGC CAA GAG CCC TGG TCC TAT-3′
	R: 5′-TGG AGG TTC CGT TTG CTG AGT-3′

Fibronectin	F: 5′-CAG TGG GAG ACC TCG AGA AG-3′
	R: 5′- CAA AGA CTA CAA GGC TCC CT-3′

Map2	F: 5′- AAC CCT TTG AGA ACA CGA CA-3′
	R: 5′-TCT TTC CGT TCA TCT GCC A-3′

Mash1	F: 5′-CCA GTT GTA CTT CAG CAC C-3′
	R: 5′-TGC CAC TTT GAG TTT GGA C-3′

NCAD	F: 5′-ACA GTG GCC ACC TAC AAA GG-3′
	R: 5′- GTA ATA GTT GGG GTA GAG CC-3′

NCAM	F: 5′-CAG CCA GCA GAT TAC AAT GC-3′
	R: 5′-TGG CTG GGA ACA ATA TCC AC-3′

Nestin	F: 5′-CTG GAG CAG GAG AAA CAG G-3′
	R: 5′-TGG GAG CAA AGA TCC AAG AC-3′

NeuroD1	F: 5′- CCA CGG ATC AAT CTT CTC AG-3′
	R: 5′-CAT GAT GTG AAT GGC TAT CG-3′

Olig2	F: 5′-GGT AAG TGC GCA ATG CTA AGC TGT-3′
	R: 5′-TAC AAA GCC CAG TTT GCA ACG CAG-3′

Pax6	F: 5′-ATG TGT GAG TAA AAT TCT GGG CA-3′
	R: 3′-GCT TAC AAC TTC TGG AGT CGC TA-3′

Sox1	F: 5′-AAT TTT ATT TTC GGC GTT GC-3′
	R: 5′-TGG GCT CTG TCT CTT AAA TTT GT-3′

Sox2	F: 5′-CCC AGC AGA CTT CAC ATG T-3′
	R: 5′-CCT CCC ATT TCC CTC GTT TT-3′

Vimentin	F: 5′-GAG AAC TTT GCC GTT GAA GC-3′
	R: 5′- CTA ACG GTG GAT GTC CTT CG -3′

18S rRNA	F: 5′-CGG CTA CAT CCA AGG AA-3′
	R: 5′-GCT GGA ATT ACC GCG GCT-3′

Dcx: doublecortin; Map2: microtubule associated protein 2; NeuroD1: neuronal differentiation 1; NCAD: neural cadherin; NCAM: neural cell adhesion molecule; Olig2: oligodendrocyte transcription factor 2; Pax6: paired box protein 6; Sox: sex-determining region Y-box.

**Table 2:** Antibodies used in this study.

Antibody	Dilution	Application	Company
CD95	1 : 100	FACS	BD Biosciences
CD105	1 : 100	FACS	BD Biosciences
CD54	1 : 100	FACS	BD Biosciences
CD56	1 : 100	FACS, IF	BD Biosciences
Nestin	1 : 200	IF	Millipore
Pax6	1 : 500	IF	Covance
Sox2	1 : 200	IF	Millipore
*β*III-Tubulin	1 : 1,000	IF	Covance

FACS: fluorescence-activated cell sorting; IF: immunofluorescence staining; Pax6: paired box protein 6; Sox2: sex-determining region Y-box 2.
