# Supplementary material for: Characterization of Neurogenic Potential of Dental Pulp Stem Cells Cultured in Xeno/Serum-Free Condition: In Vitro and In Vivo Assessment
Source: Stem Cells Int. 2016 Sep 5;2016:6921097. doi: 10.1155/2016/6921097 (PMC5027310; doi:10.1155/2016/6921097)
Supplement: Supplementary file 1 — Supplemental Figure 1. qRT-PCR measurements of NSC and MSC markers in the individual FBS (F1, F2, and F3, white bars) and NSC (N1, N2, and N3, black bars) groups at passage 5, and human BM-MSC (BM, dotted bar) and human fetal neural stem cells (FN, dashed bar) as controls. Supplemental Figure 2. Immunocytofluorescence findings of FBS and NSC medium cells at passage 5 just before the procedure for differentiation to neurons. Supplemental Figure 3. Immunohistofluorescence findings of rat brain tissues at 1-week post-transplantation of FBS medium cells. [file 6921097.f1.doc]

**Supplemental Figure 1.**

qRT-PCR measurements of NSC and MSC markers in the individual FBS (F1, F2, and F3, white bars) and NSC (N1, N2, and N3, black bars) groups, and human BM-MSC (BM, dotted bar) and human fetal neural stem cells (FN, dashed bar) as controls. The results are displayed relative to expression in the FBS medium cells via calculation using the ΔΔCT and 2-(ΔΔCT) methods and normalized to 18s ribosomal RNA expression.

**Supplemental Figure 2.**

Immunocytochemical findings of FBS and NSC medium cells just before the procedure for differentiation to neurons. βIII-tubulin positive cells were stained in red, and nuclear counter-staining was performed with DAPI (blue). Scale bars = 100 µm

**Supplemental Figure 3.**

Immunohistochemical findings of rat brain tissues at 1-week post-transplantation of FBS medium cells. The tissues were stained with human nuclei (HN, green, e), GFAP (red, f), nestin (green, b) and sox2 (red, c), and nuclear counter-staining was performed with DAPI (blue). b-d and e-g images are magnified images of yellow-line boxes in a and d images respectively. White scale bars = 100 µm, yellow scale bar=20 µm.
